# Value‐based pricing: Toward achieving a balance between individual and population gains in health benefits

**DOI:** 10.1002/cam4.2694

**Published:** 2019-11-11

**Authors:** Ambica Parmar, Tina Jiao, Ronak Saluja, Kelvin K. W. Chan

**Affiliations:** ^1^ Odette Cancer Centre Sunnybrook Health Sciences Centre Toronto ON Canada; ^2^ Sunnybrook Research Institute Toronto ON Canada; ^3^ Department of Medicine University of Toronto Toronto ON Canada; ^4^ Canadian Centre for Applied Research in Cancer Control Toronto ON Canada

**Keywords:** drug costs, health economics, health expenditures, health policy, reimbursement mechanisms

## Abstract

**Objectives:**

Value‐based pricing of oncology drugs provides a best estimate for the price of a drug, as it relates to the benefits it provides for individual patients. To date, the impact of value‐based pricing to reference cost‐effectiveness thresholds (λ) on individual and population‐level health benefits remains uncharacterized. The current study examined the potential benefits of value‐based pricing by quantifying the incremental net health benefit (INHB) of publicly funded oncology drugs, if funding occurred at manufacturer‐submitted price without value‐based pricing.

**Methods:**

Pan‐Canadian Oncology Drug Review (pCODR) submissions were reviewed to identify eligible drug indications from which final economic guidance panel reports were reviewed for incremental costs (ΔC) and quality‐adjusted life‐years (ΔQALY) from manufacturer‐submitted, pCODR lower‐limit (pCODR‐LL) and upper‐limit (pCODR‐UL) re‐analyzed estimates. Annual number of cases in Ontario for each drug indication was obtained from population databases. Annual QALY gain per drug indication was determined by (ΔQALY × cases). Population QALY gain/loss in the absence of value‐based pricing to reference λ was estimated by the INHB: (INHB = [ΔQALY − (ΔC/λ)] × cases).

**Results:**

In total, 34 drug indications (4629 cases) were identified. Annual gain in QALYs for the funded drug indications using manufacturer, pCODR‐LL, and pCODR‐UL estimates was 1851, 1617, and 1301, respectively. At a λ $100 000/QALY, funding in the absence of value‐based pricing resulted in loss of 2311, 2519, and 2604 QALYs. This would result in a provincial net annual loss of 460, 902, and 1303 QALYs.

**Conclusions:**

Despite an annual gain in QALY per funded drug indication, a net loss in QALY for the province, in the absence of value‐based pricing, was demonstrated. Supportive evidence exists for value‐based pricing toward the promotion of health benefits for the greater population.

## Graphical abstract

Value‐based pricing allows for more accurate estimates of the generated value for a novel therapeutic. However, the impact of drug funding at value‐based prices, as compared to manufacturer‐submitted prices, for contemporary cancer therapeutics remains under‐characterized. By quantifying the incremental net health benefit and incremental net monetary benefit, this study revealed overall population‐level net gains with drug funding at value‐based pricing to various reference cost‐effectiveness thresholds.

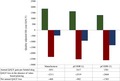

## 1 INTRODUCTION

The cost of cancer care has received increasing attention given the rising healthcare expenditure across the cancer spectrum in North America.^1^, ^2^, ^3^ A major contributing component to this notable rise in healthcare expenditure is the higher costs of modern cancer‐directed therapies, many of which cost upwards of $100 000 US dollars annually.^4^, ^5^ As many of these novel therapies are associated with only modest improvements in survival, careful consideration of the incremental cost per health outcome gained of these novel treatments is required prior to investment.^6^

Value‐based pricing offers a method to provide the best estimate for the price of a drug as it relates to the benefits it provides for the individual patients it is applied to.^7^, ^8^, ^9^ In this setting, the value of a novel therapeutic is defined as the incremental benefit an intervention offers, as compared to a reference standard of care. As such, value‐based pricing is intended to regulate prices of drugs by providing more accurate representation of the cost per added value. Accordingly, a better estimate of a therapy's value‐based price may help mitigate healthcare “over‐spending” on treatments that offer minimal gains to patients and in fact, detract health benefits from the population by diverting expenditure from other sectors of the healthcare system.^7^, ^9^

Methods utilized in the derivation of value‐based prices include cost‐effectiveness analyses (CEA), which evaluate the additional benefit of a novel therapeutic as compared to a reference standard.^7^, ^10^ Classically, the generation of an incremental cost‐effectiveness ratio [ICER=incremental cost (ΔC)/incremental effectiveness (ΔE)] with effectiveness represented by quality‐adjusted life‐years (QALY) has allowed for a standardized comparison of the therapy of interest to a comparator treatment.^11^ Using this approach, a treatment is deemed cost‐effective if the ICER is below a cost‐effectiveness threshold (λ), which currently does not hold a universally accepted value.^12^, ^13^, ^14^ Although the ICER provides information on the comparative cost‐benefit implications of an intervention, little information toward the population‐level benefits can be determined from the ICER.^15^

Application of the net‐benefit framework with generation of a net health benefit $\left(\text{NHB}=E\left(\text{QALY}\right)-\frac{C\left(\$\right)}{\lambda \left(\$/\text{QALY}\right)}\right)$ and net monetary benefit $\left(\text{NMB}=\left[E\left(\text{QALY}\right)\times \lambda \left(\$/\text{QALY}\right)\right]-C\left(\$\right)\right)$ is often considered toward assessing population‐level implications of health investment decisions.^16^, ^17^, ^18^ For comparative analyses, the incremental net health benefit (INHB) is then the difference in NHB between the new and standard treatment, defined as follows: $\text{INHB}=\Delta E\left(\text{QALY}\right)-\frac{\Delta C\left(\$\right)}{\lambda \left(\$/\text{QALY}\right)}$.^16^ If the INHB is positive, investing in the new treatment results in a net gain in QALY relative to the standard, whereas a negative INHB suggests a net loss in QALY relative to the standard.^16^ To evaluate potential gains or losses in monetary terms, the incremental net monetary benefit (INMB) can be utilized, defined as follows: $\text{INMB}=\left[\Delta E\left(\text{QALY}\right)\times \lambda \left(\$/\text{QALY}\right)\right]-\Delta C\left(\$\right)$.^17^ Thus, in addition to providing a measure of benefit for a new therapy, derivation of the INHB and INMB provides a standardized measure to compare population‐level gains or losses that may result from treatment investment, as compared to a relative cost‐effectiveness threshold.

Utilizing these methods, healthcare jurisdictions can evaluate the value‐based price of a novel therapeutic and, in comparison to an implicit or explicit cost‐effectiveness threshold, make decisions for therapy investment. According to this approach, a value‐based price will be less than or equal to an implicit or explicit cost‐effectiveness threshold.^19^ In some jurisdictions, evaluation of a therapy's value‐based price may be used to assist with drug price negotiations. In these circumstances, drug price negotiations to reduce the price from the manufacturer‐submitted to a value‐based price are done to ensure the most appropriate cost for benefit is achieved. In doing so, drug price negotiations will limit “over‐spending” on therapies with limited value, thereby generating savings in QALY or monetary benefits that can then be applied to other sectors of the healthcare system.

To date, there is limited evidence to inform on the individual and population‐level health benefits of contemporary cancer therapeutics, if value‐based pricing was adopted. As such, the current study aimed to evaluate the INHB and INMB, with value‐based pricing to reference cost‐effectiveness thresholds, for contemporary cancer therapeutics that have positive funding recommendations in Canada. The objectives for this study were to (a) quantify the total INHB from the Canadian public‐payer's perspective to characterize the potential gain/loss in QALY with drug funding at value‐based prices to various reference cost‐effectiveness thresholds, and (b) quantify the INMB with value‐based prices to various reference cost‐effectiveness thresholds.

## 2 METHODS

Institutional ethics approval from Sunnybrook Health Sciences Centre's Research and Ethics Board was obtained prior to study commencement.

### 2.1 Selection of drug reviews

All oncology drug indications submitted from July 13, 2011 (ie, the inception of the pan‐Canadian Oncology Drug Review [pCODR]) to September 25, 2018 were identified by a systematic review of the pCODR drug reviews from the Canadian Agency for Drugs and Technologies in Health (CADTH) website (https://cadth.ca/pcodr). Reviews were excluded if there was no publicly available Final Economic Guidance Report or Provincial Summary File and/or if they did not disclose best estimates of manufacturer‐submitted or pCODR re‐analyzed values of incremental cost (ΔC) and/or incremental effectiveness (ΔE) in their Final Economic Guidance Report. In the setting of manufacturer resubmission with no alteration in patient indication, only the submission that resulted in a funding decision was included to avoid duplication. The funding statuses of the remaining reviews were identified using the Provincial Funding Summary files. Based upon the funding status, only indications that were funded prior to March 2018 were included to ensure the most up‐to‐date case numbers were obtained. Furthermore, to ensure accuracy in case number identification, only drugs with unique, distinguishable indications were included in the final analysis.

### 2.2 Data extraction

The extracted baseline characteristics of the drug reviews including the generic drug name, pCODR number, strength, route of administration, cancer type, indication, pCODR Expert Review Committee (pERC) final recommendation (ie, one of recommend funding, recommend funding with conditions, do not recommend funding), and date of final recommendation were independently extracted by two reviewers (TJ and RS). Discrepancies were resolved by consensus or through consultation with a third reviewer (KC). From the Provincial Funding Summaries taken from the pCODR/CADTH website, the extracted data included the funding status, funding date, and funding criteria for Ontario. From the Final Economic Guidance Report, the economic values (ICER, ΔC, and ΔE), and the drug comparisons used to establish them were extracted. The economic values included the manufacturer‐submitted, and the lower‐ and upper‐limit best estimates of the pCODR re‐analyzed model, as generated by the Economic Guidance Panel's (EGP) multiple scenario analyses to address uncertainties and limitations in the submission. In cases where multiple sets of economic values were reported in a single drug review due to multiple comparisons or indications, the comparison relevant to the funded indication was chosen. In cases where only one set of pCODR re‐analyzed values was given, instead of a lower‐upper range, the set was extracted as both the lower and upper estimates for analytic purposes. Table S1 summarizes the extracted economic values for the included drugs.

For each drug indication funded by Ontario, the numbers of new cases in the fiscal year of 2017‐2018 were obtained by reviewing the New Drug Funding Program (NDFP) and Ontario Drug Benefit (ODB) databases. With a population over 14 000 000, Ontario makes up 39% of the Canadian population making it the most populous province in Canada.^20^ Accordingly, Ontario data provide the highest volume of oncology patients, allowing for representative Canadian data. The NDFP database contains information about the exact indication for use of all publicly funded intravenous drug therapies administered at cancer facilities in Ontario. In contrast, the ODB database, inclusive of orally administered cancer therapeutics, does not contain reliable indication information. Accordingly, drugs found in the ODB that had more than one listed indication were excluded from the analysis.

### 2.3 Statistical analysis

Descriptive statistics were utilized to summarize the characteristics of the included drug reviews. The annual QALY gain associated with a drug indication was estimated by applying the following formula: [ΔE(QALY) × new cases in fiscal year]. The QALY gain/loss per patient for each funded indication was calculated by applying the following formula for INHB: $\left[\text{INHB}=\Delta E-\frac{\Delta C\left(\$\right)}{\lambda \left(\$/\text{QALY}\right)}\right]$. Population QALY gain/loss for each indication was calculated by multiplying the QALY gain/loss per patient by the number of new cases for the indication, in the fiscal year of 2017‐2018 in Ontario: (Population $\text{INHB}=\left[\Delta E\left(\text{QALY}\right)-\frac{\Delta C\left(\$\right)}{\lambda \left(\$/\text{QALY}\right)}\right]$ × new cases in fiscal year). Utilizing these formulas, a positive INHB results in a net gain in QALY, whereas a negative INHB suggests a net loss in QALY. As such, a net loss in QALY represents a population loss in health benefits. These two formulas were applied to manufacturer‐submitted, lower‐ and upper‐limit pCODR re‐analyzed values using threshold‐defined value‐based prices of $50 000/QALY, $100 000/QALY, and $150 000/QALY. Annual population gain/loss in monetary terms was calculated by applying the following formula: (Population $\text{INMB}=\left[\Delta E\left(\text{QALY}\right)\times \lambda \left(\frac{\$}{\text{QALY}}\right)\right]-\Delta C\left(\$\right)$ × new cases in fiscal year) at threshold‐defined value‐based prices of $50 000/QALY, $100 000/QALY, and $150 000/QALY. All costs were inflated to 2018 Canadian dollars.^21^

## 3 RESULTS

**Figure 1:**
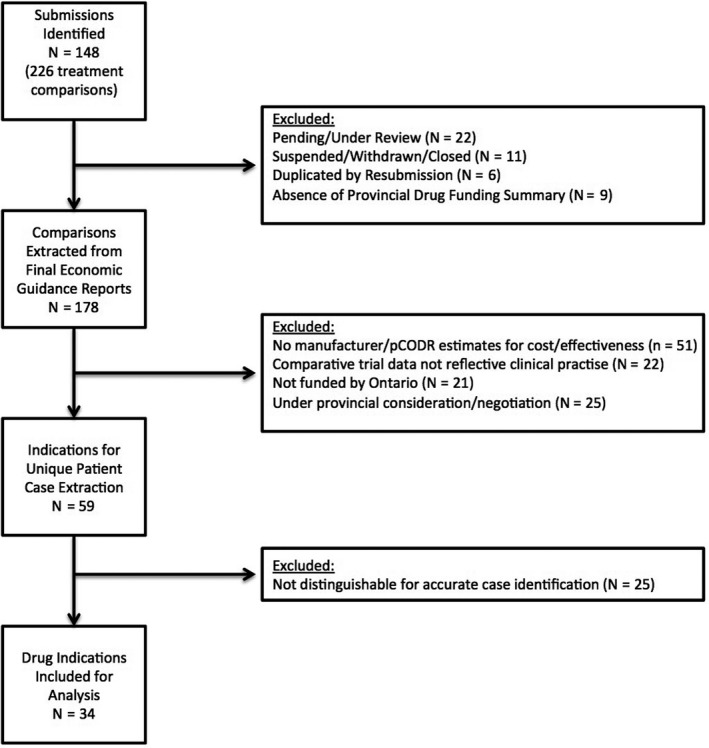
Selection of drug reviews. Flow diagram depicting the systematic review and selection process for included drug reviews as per inclusion and exclusion criteria. pCODR, pan‐Canadian Oncology Drug Review

In total, 148 submissions from pCODR/CADTH were identified from which 34 indications for drug reviews were included based upon the inclusion criteria (Figure 1). The most common cancer types of the included drug reviews were melanoma (18%), leukemia (15%), lung (15%), and lymphoma (15%). A total of 4629 new cases identified in the fiscal year of 2017‐2018 were included in the analysis. Table 1 outlines the characteristics of the included drug reviews and the distribution of new cases.

**Table 1:** Characteristics of the included drug reviews

Variable	Number of reviews (%)	Number of new cases 2017/2018 (%)
Route of administration		
Oral	8 (24)	484 (10)
Intravenous	26 (76)	4145 (90)
Cancer type		
Breast	3 (9)	378 (8)
Endocrine	1 (3)	25 (1)
Gastrointestinal	3 (9)	1120 (24)
Genitourinary	1 (3)	172 (4)
Gynecology	2 (6)	157 (3)
Head and neck	1 (3)	24 (1)
Leukemia	5 (15)	243 (5)
Lung	5 (15)	1107 (24)
Lymphoma	5 (15)	685 (15)
Myeloma	1 (3)	153 (3)
Other	1 (3)	13 (1)
Skin and melanoma	6 (18)	552 (12)
Indication		
First line	18 (53)	2629 (57)
Second line or beyond	15 (44)	1997 (43)
Not specified	1 (3)	3 (<1)

**Figure 2:**
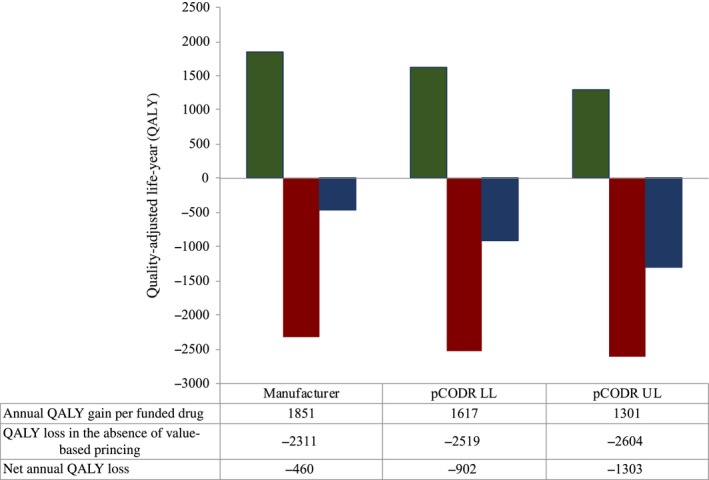
Gain/Loss in QALY for Ontario, Canada, in fiscal 2017‐2018 using manufacturer‐submitted, pCODR lower‐limit and upper‐limit economic estimates in the absence of value‐based pricing to a reference cost‐effectiveness threshold of $100 000/QALY. Graphical representation of the annual gain in QALY with funded drug indications, as well as loss in QALY in the absence of value‐based pricing to a reference cost‐effectiveness threshold of $100 000/QALY. LL, lower limit; pCODR, pan‐Canadian Oncology Drug Review; QALY, quality‐adjusted life‐year; UL, upper limit

**Figure 3:**
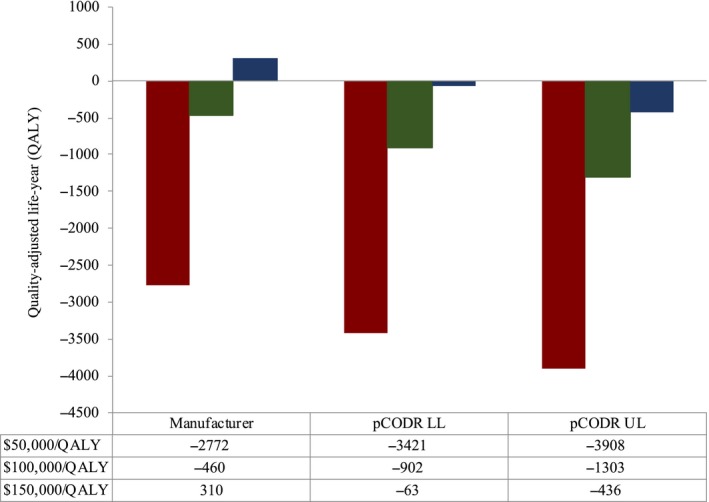
Annual net gain/loss in QALY for Ontario, Canada in fiscal 2017‐2018 in the absence of value‐based pricing to reference cost‐effectiveness thresholds of $50 000/QALY, $100 000/QALY, and $150 000/QALY. Graphical representation of the net annual gain/loss in QALY for funded drug indications in Ontario, Canada using manufacturer‐submitted, pCODR lower‐ and upper‐limit economic estimates. LL, lower limit; pCODR, pan‐Canadian Oncology Drug Review; QALY, quality‐adjusted life‐year; UL, upper limit

The total QALY lost in Ontario, Canada when evaluating included drug reviews by manufacturer‐submitted, pCODR re‐analyzed upper‐ and lower‐limit estimates for the 2017‐2018 fiscal year are represented in Figures 2 and 3. The annual QALY gain $\left(\Delta E\left(\text{QALY}\right)\times \;\text{new cases}\right)$ for funded drug indications using manufacturer‐submitted, pCODR re‐analyzed lower‐ and upper‐limit effectiveness estimates were 1851, 1617, and 1301 respectively. At a cost‐effectiveness threshold of $100 000/QALY, drug funding in the absence of value‐based pricing using manufacturer‐submitted, pCODR re‐analyzed lower‐ and upper‐limit cost estimates resulted in annual QALY losses of 2311, 2519, and 2604, respectively. This resulted in a net annual QALY loss of 460, 902, and 1303, respectively. (Figure 2) Accordingly, although the patients within these drug indications derived benefits (ie, gain in QALY), the overall net loss in QALY demonstrates a population loss in health benefits, whereby investment in an alternative health technology at a lower cost could have resulted in QALY gains.

The total net QALY lost for Ontario at a threshold of $50 000/QALY was 2772, 3421, and 3908, respectively. At a threshold of $150 000/QALY, QALY loss of 63 and 436 were demonstrated when examined with the pCODR upper‐ and lower‐limit re‐analyzed estimates, but a QALY gain was noted using the manufacturer‐submitted price. (Figure 3) Accordingly, a higher cost‐effectiveness threshold was required to see an improvement in the QALY loss, in the absence of value‐based pricing.

**Figure 4:**
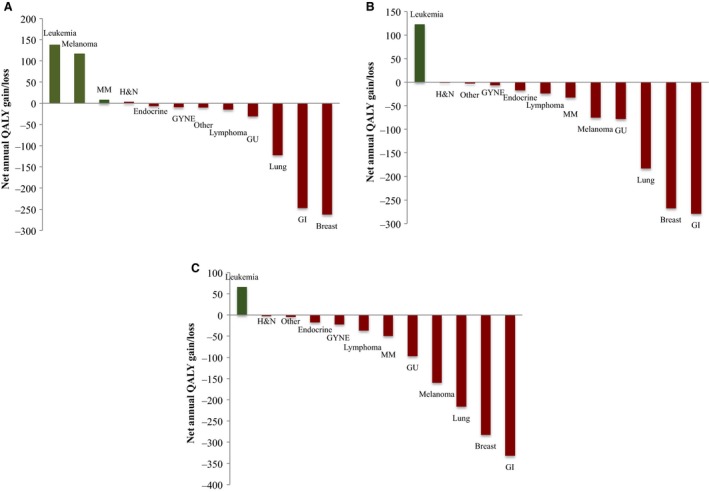
Net annual QALY gain/loss for Ontario, Canada in fiscal 2017‐2018 per cancer indication in the absence of value‐based pricing to a reference cost‐effectiveness threshold of $100 000/QALY. Graphical representation of the net annual gain/loss in QALY for funded drug indications in Ontario, Canada per cancer disease site utilizing: (A) manufacturer‐submitted economic estimates; (B) pCODR lower‐limit re‐analyzed economic estimates; (C) pCODR upper‐limit re‐analyzed economic estimates. GI, gastrointestinal cancers; GU, genitourinary cancers; GYNE, gynecological cancers; H&N, head and neck cancers; LL, lower limit; MM, multiple myeloma; Other, sarcoma, pCODR, pan‐Canadian Oncology Drug Review; QALY, quality‐adjusted life‐year; UL, upper limit

When examined by drug indication, a few trends were noted. Across manufacturer‐submitted, pCODR lower‐ and upper‐limit re‐analyzed estimates, a consistent QALY gain was seen with drugs for leukemia, when analyzed to a reference cost‐effectiveness threshold of $100 000/QALY. Within the same analysis, a consistent loss in QALY was noted with drugs used in breast, lung, gastrointestinal, and genitourinary indications, with the most prominent losses seen within cancer disease sites of gastrointestinal and breast. Figure 4 represents the QALY loss/gain per drug indication.

**Figure 5:**
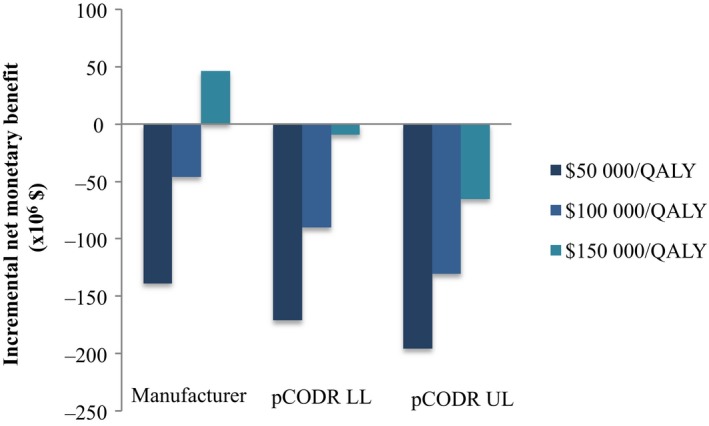
Incremental net monetary benefit for Ontario, Canada in fiscal 2017‐2018 in the absence of value‐based pricing to reference cost‐effectiveness thresholds of $50 000/QALY, $100 000/QALY, and $150 000/QALY. Graphical representation of the net monetary gain/loss in 2018 Canadian dollars for funded drug indications in Ontario, Canada at manufacturer‐submitted, pCODR lower‐ and upper‐limit economic estimates. LL, lower limit; pCODR, pan‐Canadian Oncology Drug Review; QALY, quality‐adjusted life‐year; UL, upper limit

To determine the impact in monetary terms, the INMB for Ontario, Canada at reference thresholds of $50 000/QALY, $100 000/QALY, and $150 000/QALY were estimated (Figure 5). Using the manufacturer‐submitted, pCODR lower‐ and upper‐limit re‐analyzed estimates, annual monetary loss was $46 077 354, $90 212 119, and $130 363 955, respectively, at a threshold of $100 000/QALY. When analyzed with a threshold of $150 000/QALY, the annual monetary loss in the absence of value‐based pricing was lower, with demonstration of net monetary benefit when analyzed by the manufacturer‐submitted estimates (Figure 5).

## 4 DISCUSSION

Through our review of oncology drug submissions in Canada, we described a loss in QALY and monetary loss, if drug funding occurred in the absence of value‐based pricing, in reference to various cost‐effectiveness thresholds. The results of our study demonstrate population losses in health benefits with drug funding at manufacturer‐submitted prices, from the perspective of the Ontario health care system. Given these population‐level losses in QALY represent potential budgetary diversions from other healthcare sectors, the results of this study highlight the need for measures to improve pricing for novel cancer therapeutics to promote both individual and population‐level health benefits.

The negative population‐level implications of “over‐spending” on cancer therapeutics have previously been demonstrated through an analysis of the Cancer Drugs Fund (CDF) in the United Kingdom (UK). The CDF was a program established by the UK government with the aim of improving access to cancer drugs that were publicly unavailable due to the absence of final National Institute for Health and Care Excellence (NICE) technology appraisals or lack of positive recommendations through NICE appraisals.^22^ Although the intent of the CDF was to improve timely access to cancer therapeutics, the high costs accrued by this program, generated discussion as to the impact the CDF was having for health benefits.^22^, ^23^, ^24^ In a policy review of the CDF‐approved indications, Aggarwal et al demonstrated a median overall survival (OS) benefit of only 3.1 months across the 47 approved cancer therapeutics.^23^ However, despite these modest incremental survival gains for the approved indications, the CDF budget has experienced regular increases in its annual budget from £200 million in 2013 to over £1 billion in 2016.^25^ With such significant spending on a small proportion of cancer therapeutics, the impact of this program on population‐level health benefits becomes critical to appraise. In a similar analysis to the current study, Claxton characterized the individual and population‐level health benefits achieved through the CDF, given a median OS of 3 months and annual spending over £230 million.^24^ In keeping with the current findings, although there was generation of over 3000 QALYs for the individuals for whom the approved drug indications applied, the significant cost associated with the approved therapeutics generated a loss of over 17 000 QALYs, representing budgetary diversions away from other sectors of the healthcare system.^24^ These results highlight the significant trade‐off in health benefits that can occur to obtain timely drug access through funding at manufacturer‐based pricing. However, unlike in the current study, Claxton's analysis was based upon crude assumptions of incremental gains in QALY with funded CDF drugs, lacking the precision of established gains in QALY per drug indication, as utilized in the current study. Furthermore, although Claxton's analysis included the total annual number of patients funded by the CDF, the author could not characterize the exact proportion of patients receiving each funded drug indication. As our study demonstrated considerable variation in QALY gain/loss per drug indication and disease site, this absence of granular data for the proportion of patients within each drug indication can lead to inaccurate representation of both individual and population‐level benefits. Accordingly, the current analysis offers a contemporary, granular assessment as to precise estimates for the population‐level consequences of drug funding in the absence of value‐based pricing. Nevertheless, the lessons from the CDF complement the current analysis' conclusions that value‐based prices are needed to reduce the negative impact that drug funding of costly drugs can have on population‐level health benefits.^24^

Drug price negotiations to reduce prices to implicit or explicit cost‐effectiveness thresholds are a recognized approach to reduce healthcare expenditure on pharmaceuticals in certain healthcare jurisdictions. For instance, in Canada drug‐funding recommendations are made through a coordinated process with the establishment of collaborative programs, namely the Common Drug Review (CDR) and pCODR, for all provinces outside of Quebec, and the Institute national d'excellence en santé et en services sociaux (INESSS) for Quebec.^26^, ^27^ These bodies review submissions from pharmaceutical manufacturers to provide funding recommendations in reference to an estimated value‐based price for a novel therapy. Further, to allow for coordinated price negotiations, the Pan‐Canadian Pharmaceutical Alliance (pCPA) was established in an effort to improve the consistency of drug coverage across jurisdictions.^28^, ^29^ Overall, the pCPA aims to improve drug costs and achieve consistent pricing across the Canadian provinces and territories.^29^ Through these efforts, drug price negotiations to value‐based prices can generate savings in QALY or monetary benefits that can then be applied to other sectors of the health care system. As such, with manufacturer‐based pricing that commonly exceeds value‐based prices, the current analysis would also imply value for drug price negotiation efforts. However, the actual impact of drug‐price negotiations for the generation of population‐level health benefits in Canada remains uncertain, given current processes for price negotiations are kept confidential.

Upon analysis by the INMB, a net monetary loss was also demonstrated in the absence of value‐based pricing. Annual net losses of greater than $40 000 000 at a cost‐effectiveness threshold of $100 000/QALY represents a significant impetus to pursue methods to reduce current drug therapy costs toward value‐based prices. An improvement in drug price costs not only has the potential to improve budgetary constraints in a publicly funded universal health care system but there is an additional financial benefit in other healthcare systems, such as the US, particularly in situations where patients incur out‐of‐pocket costs.^30^, ^31^ With upwards of 10% of cancer care costs paid out‐of‐pocket, the financial burden of cancer care is felt, even among insured US patients.^32^ These high costs for patients are counterproductive to the goal of improving patient outcomes, given the potential for detriment to patient well‐being and the possibility of non‐compliance and adherence given patients' inability to tolerate the financial burden.^32^, ^33^ Similarly, the financial well‐being of health benefit programs necessitates methods of maintaining cost‐effective purchasing.^31^ Annual spending for prescription drug expenditures across Medicare and Medicaid is estimated at over $130 billion US dollars with a projected increase in expenditure over the next decade.^34^ With healthcare spending in the US growing faster than the gross domestic product (GDP), these rising costs threaten the financial sustainability of these programs.^34^ As such, methods to reduce costs are warranted to promote and sustain these health benefit programs.^31^ This understood need has led to significant support for the use of drug price negotiations in the US as a cost‐saving measure.^30^, ^31^, ^35^, ^36^, ^37^

A challenge in the application of value‐based pricing is the absence of a well‐accepted universal cost‐effectiveness threshold. Estimates for appropriate cost‐effectiveness thresholds have been debated with considerable global variation in thresholds adopted for drug funding decisions.^14^, ^38^, ^39^, ^40^, ^41^ In the UK and Ireland, explicit thresholds of cost‐effectiveness have been defined, whereas in Canada and the US no explicit threshold is recognized.^41^ Historically referenced as US$50 000/QALY, recently the Institute for Clinical and Economic Review has recommended an acceptable cost‐effectiveness threshold in the US as ranging from US$50 000 to 175 000/QALY.^42^ In Canada, implicit thresholds of CAD$20 000‐100 000/QALY have previously been suggested.^43^ The utilization of these cost‐effectiveness thresholds has largely been as a guide for drug funding decisions. However, their arbitrary derivation based largely upon the estimated need (ie, “demand‐side”) for health interventions, with a lack of consideration for resource constraints has generated discussion as to how these thresholds may negatively impact population‐level health benefits.^44^, ^45^

Empirical thresholds (ie, “supply‐side”) derived from country‐specific measurements of healthcare outcomes and resource constraints may offer a more precise method to derive cost‐effectiveness thresholds through a better understanding of the threshold of cost which would result in negative healthcare resource displacement (ie, loss in QALY).^44^, ^45^ Methods to estimate country‐specific empirical ICER have been published, frequently reflecting empirical thresholds that are lower than previously discussed “demand‐side” thresholds.^44^, ^45^, ^46^, ^47^ The differences noted in empirical and “demand‐side” cost‐effectiveness thresholds highlight the concern with universal adoption of arbitrary higher thresholds. Consistently applying higher cost‐effectiveness thresholds will threaten population‐level health benefits and long‐term financial sustainability, as demonstrated in the current analysis with a lower gain in INHB and INMB through value‐based pricing at higher cost‐effectiveness thresholds. In Canada, there is now growing discussion toward the possibility of adopting empirical “supply‐side” cost‐effectiveness thresholds to reduce the potential disinvestment that may be incurred through funding of high‐cost drugs.^48^ However, recognizing the significant costs associated with many novel therapeutics, the applicability of this approach remains to be seen. Nevertheless, it is evident a balance must be achieved to ensure utilized thresholds demonstrate applicability to the current landscape of health technologies while reflecting an appropriate measure of health benefit.^14^, ^38^

An additional challenge in current value‐based pricing is in the measurement of therapeutic value. In both traditional CEA and evaluation of INHB, effectiveness is measured through the generation of a QALY. However, there are additional considerations when determining the value of a therapy that are not captured by these measures. These include the following: (a) disease‐specific considerations such as disease burden or severity of disease; (b) patient‐specific considerations including preferences in toxicity profiles, therapeutic choice, and caregiver implications; and (c) technological considerations including contribution to innovation. As these additional disease‐specific considerations are not captured in the measure of QALY, using CEA and INHB oversimplifies the value for a given therapy. As a result, use of a CEA has the potential to lead to inaccurate estimates of a therapy's value‐based price. Multi‐criteria decision analysis (MCDA) offers a promising framework to incorporate these considerations into a comprehensive value assessment to inform value‐based pricing.^49^ However, some concerns exists with the reliability of MCDA to determine value given the complex methodology required.^42^ Thus, further validation and standardization of this methodology is required prior to universal use of this framework in the current application. Nevertheless, health‐technology assessment groups routinely take the influence of these additional attributes of a therapy into consideration when making recommendations for drug‐funding decisions.^50^, ^51^

This study has several strengths. Through a comprehensive review of all pCODR drug submissions since inception, the data acquired create a strong body of evidence to investigate the potential impact of value‐based pricing on population‐level INHB and INMB. Also, evaluation of the INHB and INMB at various reference threshold‐defined value‐based prices allowed for a better understanding of the influence of these arbitrary thresholds on the derivation of population‐level benefits. A notable limitation of this study is, although this was a comprehensive review of Canadian drug approvals, our inclusion criteria limited the number of included drug indications. However, this was necessary to limit duplication in the analysis given the limitations of drug indication information in the oral drug database in Ontario. The lack of drug indication information is likely a common limitation in oral drug claims databases in most jurisdictions.

In conclusion, drug funding of contemporary cancer therapeutics at current manufacturer pricing has the potential to lead to significant losses in population NHB. As drug costs continue to rise, a strategic focus on measures to improve costs for these therapies is going to be imperative for the global benefit of our healthcare system. Utilization of value‐based pricing to appropriate reference cost‐effectiveness thresholds represents a viable option toward population‐level gains in health.

## AUTHOR CONTRIBUTIONS

Ambica Parmar: Formal analysis, writing‐original draft, and editing. Tina Jiao: Data curation, formal analysis, editing. Ronak Saluja: Data curation, formal analysis, and editing. Kelvin KW Chan: Conceptualization, data curation, formal analysis, and editing.
