# Supplementary material for: Value‐based pricing: Toward achieving a balance between individual and population gains in health benefits
Source: Cancer Med. 2019 Nov 11;9(1):94–103. doi: 10.1002/cam4.2694 (PMC6943176; doi:10.1002/cam4.2694)
Supplement: Supplementary file 1 [file CAM4-9-94-s001.docx]

**Supplemental Table 1.** Details of Included Systemic Therapies

| **Systemic Therapy** | **Indication** | **Manufacturer Submitted** | | | **pCODR Lower Limit** | | | **pCODR Upper Limit** | | |
| --- | --- | --- | --- | --- | --- | --- | --- | --- | --- | --- |
|  |  | **ΔC ($)** | **ΔE (QALY)** | **ICER ($/QALY)** | **ΔC ($)** | **ΔE (QALY)** | **ICER ($/QALY)** | **ΔC ($)** | **ΔE (QALY)** | **ICER ($/QALY)** |
| Arsenic Trioxide | APL – 1^st^ Line Low/Intermediate Risk | $77,955 | 1.44 | 50,192 | $82,490 | 1.44 | 53,112 | 82,490 | 1.44 | 53,112 |
| Arsenic Trioxide | APL – 1^st^ Line High Risk | $5,161 | 0.33 | 14,703 | $7,758 | 0.33 | 22,102 | $7,758 | 0.33 | 22,102 |
| Arsenic Trioxide | APL – 2^nd^ Line Relapsed/Refractory | $41,236 | 1.87 | 20,443 | $34,714 | 2.41 | 13,338 | $38,667 | 0.45 | 80,263 |
| Afatinib | Advanced NSCLC | $16,362 | 0.21 | 72,153 | $620 | 0.015 | 39,060 | $583 | 0.0026 | 211,189 |
| Bendamustine | Non-Hodgkin’s Lymphoma | $17,448 | 0.3 | 53,418 | $25,203 | 0.2459 | 94,071 | $25,203 | 0.2459 | 94,071 |
| Bendamustine | Chronic Lymphocytic Leuekemia | $25,964 | 0.52 | 52,606 | $29,927 | 0.28 | 98,321 | $29,927 | 0.28 | 98,321 |
| Bevacizumab | Advanced Cervical Cancer | $46,842 | 0.303 | 145,957 | $46,465 | 0.278 | 157,829 | $49,981 | 0.192 | 245,452 |
| Bevacizumab | Advanced Colorectal Cancer | $56,567 | 0.254 | 212,938 | $56,567 | 0.254 | 211,938 | $55,550 | 0.171 | 309,763 |
| Bevacizumab | Advanced Ovarian Cancer | $38,150 | 0.374 | 96,261 | $39,127 | 0.424 | 87,033 | $37,263 | 0.317 | 113,473 |
| Brentuximab | Hodgkin’s Lymphoma | $140,081 | 1.16 | 111,752 | $137,337 | 0.94 | 135,684 | $137,337 | 0.94 | 135,684 |
| Brentuximab | Anaplastic Large Cell Lymphoma | $102,189 | 1.17 | 81,055 | $142,731 | 1.018 | 130,498 | $142,731 | 1.018 | 130,498 |
| Cobimentinib | Advanced Melanoma | $ 164,563 | 0.495 | 317,648 | 164,563 | 0.5 | 314,268 | $164,287 | 0.367 | 426,815 |
| Crizotinib | Advanced NSCLC | $39,137 | 0.243 | 153,597 | $38,280 | 0.211 | 173,570 | $39,159 | 0.131 | 285,299 |
| Dabrafenib + Trametinib | Advanced Melanoma | $119,919 | 0.345 | 332,129 | $48,234 | 0.142 | 323,454 | $161,121 | 0.369 | 446,238 |
| Ipilimumb | Advanced Melanoma | $118,005 | 0.738 | 151,014 | $115,315 | 0.525 | 165,389 | $116,865 | 0.667 | 197,382 |
| Lenvatinib | Well-Differentiated Thyroid Carcinoma | $110,447 | 0.84 | 126,235 | $153,879 | 0.84 | 176,281 | $153,879 | 0.84 | 176,281 |
| Nab-paclitaxel | Advanced Pancreatic Cancer | $21,214 | 0.129 | 155,549 | $25,254 | 0.125 | 182,714 | $25,254 | 0.119 | 192,995 |
| Nivolumab | Advanced Head and Neck Squamous Cell Carcinoma | $31,336 | 0.456 | 67,616 | $38,318 | 0.34 | 109,743 | $34,372 | 0.23 | 145,855 |
| Nivolumab | Advanced Renal Cell Carcinoma | $65,971 | 0.481 | 131,349 | $93,577 | 0.481 | 186,312 | $93,577 | 0.37 | 242,521 |
| Nivolumab | Advanced Melanoma | $84,037 | 0.852 | 94,176 | $108,999 | 0.893 | 120,851 | $152,509 | 0.515 | 198,776 |
| Pembrolizumab | Advanced Melanoma – 1^st^ Line | $40,952 | 0.74 | 52,829 | $66,049 | 0.55 | 114,389 | $59,989 | 0.37 | 151,369 |
| Pembrolizumab | Advanced Melanoma – 2^nd^ Line | $115,689 | 0.91 | 121,843 | $105,386 | 0.17 | 586,833 | $103,644 | 0.1 | 903,678 |
| Pembrolizumab | Advanced NSCLC – 1^st^ Line | $102,632 | 0.99 | 99,392 | $112,378 | 0.96 | 111,769 | $107,966 | 0.67 | 154,273 |
| Pembrolizumab | Advanced NSCLC – 2^nd^ Line | $80,126 | 0.53 | 143,730 | $74,808 | 0.48 | 149,342 | $71,459 | 0.27 | 254,945 |
| Palbociclib | Advanced Breast Cancer | $207,381 | 0.641 | 310,007 | $182,178 | 0.647 | 295,925 | $237,549 | 0.257 | 745,785 |
| Pemetrexed | Advanced NSCLC | $39,301 | 0.25 | 143,261 | $40,449 | 0.21 | 170,272 | $41,303 | 0.22 | 173,864 |
| Pertuzumab | Advanced Breast Cancer | $131,060 | 0.505 | 238,014 | $128,495 | 0.45 | 262,263 | $109,718 | 0.332 | 303,726 |
| Pomalidomide | Multiple Myeloma | $47,509 | 0.53 | 84,476 | $74,358 | 0.53 | 132,217 | $71,381 | 0.39 | 173,430 |
| Obinutuzimab | Chronic Lymphocytic Leukemia | $31,842 | 0.975 | 30,844 | $31,729 | 0.926 | 32,369 | $30,968 | 0.587 | 49,823 |
| Ramucurimab | Advanced Gastric/ Gastro-esophageal Carcinoma | $31,225 | 0.09 | 332,628 | $40,568 | 432,159 | 0.09 | $46,038 | 0.09 | $490,437 |
| Regorafenib | Gastrointestinal Stromal Tumours | $66,177 | 0.586 | 104,660 | $61,108 | 0.407 | 143,317 | $69,320 | 0.31 | 205,299 |
| Romidepsin | Peripheral T-Cell Lymphoma | $108,310 | 0.55 | 186,253 | $111,443 | 0.4836 | 217,588 | $152,971 | 0.3207 | 387,056 |
| Siltuximab | Multi-centric Casteleman’s Disease | $308,753 | 1.44 | 204,332 | $308,753 | 1.27 | 232,663 | $204,291 | 0.3 | 648,163 |
| Trastuzumab | Advanced Breast Cancer | $59,405 | 0.433 | 127,052 | $62,451 | 0.398 | 145,403 | $62,451 | 0.398 | 145,403 |

Table Legend. Details of include systemic therapy’s economic values as extracted from the pan-Canadian Oncology Drug Review (pCODR) Expert Review Committee (pERC) final economic guidance report. ΔC: incremental cost ($, Canadian dollars); ΔE: incremental effectiveness (quality-adjusted life-years); ICER: incremental cost-effectiveness ratio; QALY: quality-adjusted life-years. All data obtained from publicly available final economic guidance reports from the Canadian Agency for Drugs and Technology in Health. [Retrieved from: <https://www.cadth.ca/>]
